# Venetoclax plus azacitidine and donor lymphocyte infusion in treating acute myeloid leukemia patients who relapse after allogeneic hematopoietic stem cell transplantation

**DOI:** 10.1007/s00277-021-04674-x

**Published:** 2021-09-27

**Authors:** Peng Zhao, Ming Ni, Dan Ma, Qin Fang, Yan Zhang, Yanju Li, Yi Huang, Ying Chen, Xiao Chai, Yun Zhan, Yan Li, Qian Kang, Mei Zhao, Min Liu, Fengqi Zhang, Shisi Huang, Shuangshuang Wen, Bo Deng, Jishi Wang

**Affiliations:** 1 Department of Hematology, The Second Affiliated Hospital of Soochow University, Suzhou, 215004 People’s Republic of China; 2 Guizhou Province Hematopoietic Stem Cell Transplantation Center, Key Laboratory of Hematological Disease Diagnostic & Treat Centre of Guizhou Province, Department of Hematology, The Affiliated Hospital of Guizhou Medical University, Guiyang, 550004 People’s Republic of China; 3 Department of Pharmacy, The Affiliated Hospital of Guizhou Medical University, Guiyang, 550004 People’s Republic of China

**Keywords:** Relapsed acute myeloid leukemia, Allo-HSCT, Venetoclax, Azacytidine, Donor lymphocyte infusion

## Abstract

This study aimed to evaluate the efficacy and safety of venetoclax plus azacitidine and donor lymphocyte infusion (DLI) in treating patients with relapsed acute myeloid leukemia (AML) after allogeneic hematopoietic stem cell transplantation (allo-HSCT). Twenty-six AML patients who relapsed after allo-HSCT were enrolled and treated with venetoclax plus azacitidine and DLI. Complete remission with incomplete recovery (CRi), partial remission (PR), and objective remission rate (ORR) were assessed, and then event-free survival (EFS) and overall survival (OS) were evaluated. Besides, adverse events were documented. Additionally, whole exome sequencing was performed in bone marrow samples. The CRi, PR, and ORR rates were 26.9%, 34.6%, and 61.5%, respectively. The median time of EFS and OS was 120 (95% CI: 71–610) days and 284.5 (95% CI: 81–610) days, respectively. The most common adverse events were hematologic system adverse events including agranulocytosis, anemia, and thrombocytopenia, while the adverse events of other systems were relatively less and milder. In addition, no serious adverse events existed. Of note, there were 6 (23.1%) patients who developed GVHD. As for gene mutation, 49 mutated genes were found, which were categorized as first-, second-, and third-class mutations, and then further analysis revealed that the first-class mutations were not correlated with EFS or OS. Additionally, the most frequent mutated genes were FLT3, CEBPA, DNMT3A, KIT, KRAS, and NRAS. Venetoclax plus azacitidine and DLI is efficient and tolerant in treating patients with relapsed AML after allo-HSCT, implying this combined therapy as a potential treatment option in the studied patients.

**Supplementary Information:**

The online version contains supplementary material available at 10.1007/s00277-021-04674-x.

## Introduction

Acute myeloid leukemia (AML) is a major type of hematological malignancies with the highest prevalence among all kinds of leukemias [1, 2]. Relapsed disease has always been a predominant challenge in AML treatment, and most of the relapsed patients are at an older age, which obviously enhances the difficulty of management [3–5]. Allogeneic hematopoietic stem cell transplantation (allo-HSCT) is an important curative therapy for the eligible AML patients; however, although treated by allo-HSCT, there are still approximately 40% patients who relapse post treatment [6]. More importantly, relapse after allo-HSCT treatment often induces a poor prognosis; however, the treatment options available for this patient group are quite limited.

**Fig. 1:**
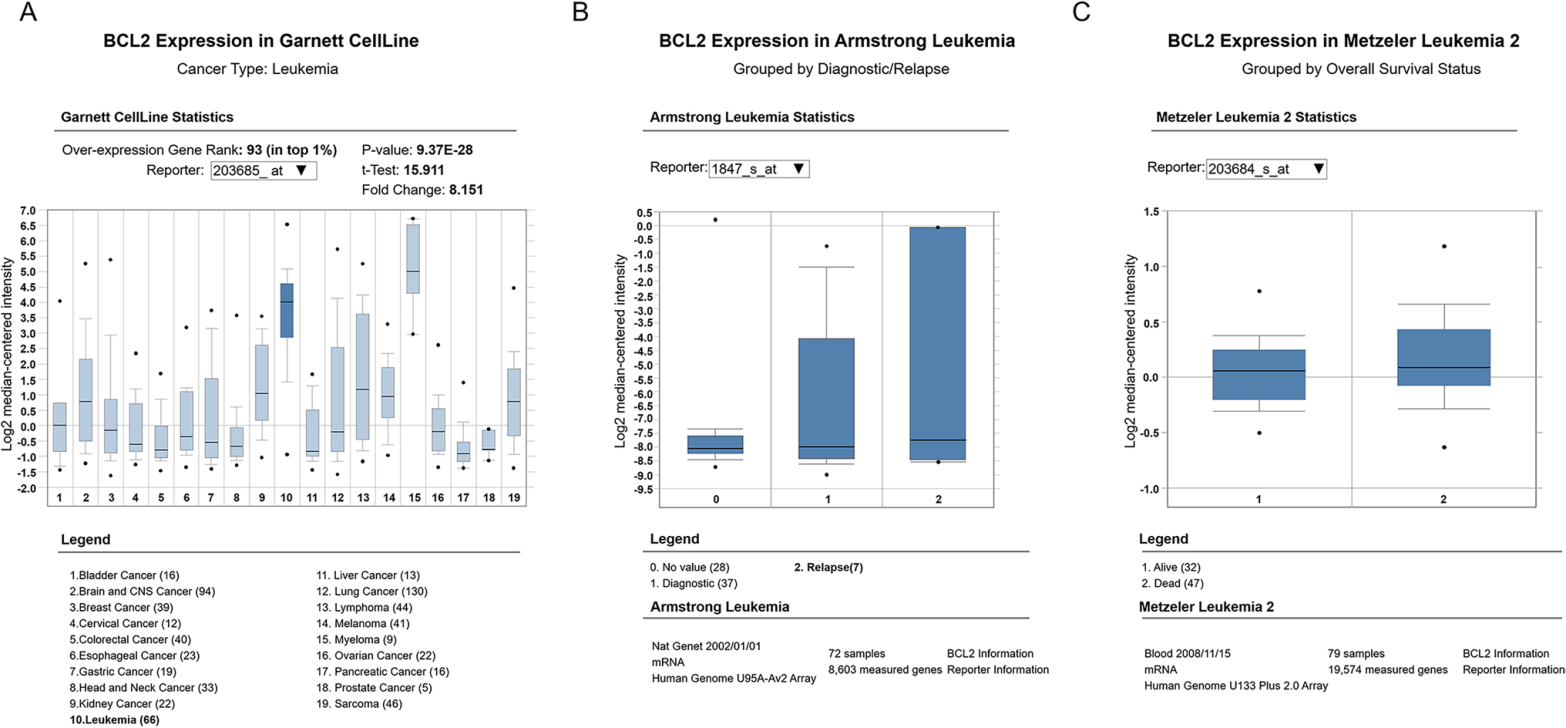
BCL2 expression in leukemia (data from Oncomine database). BCL2 expression by Garnett CellLine (**A**), BCL2 expression in Armstrong leukemia (**B**), and BCL2 in Metzeler leukemia 2 (**C**). BCL2, B-cell lymphoma 2; AML, acute myeloid leukemia

Venetoclax is a selective inhibitor of the anti-apoptosis factor B-cell lymphoma 2 (BCL2), which has been revealed to abundantly express in leukemia stem cells [7, 8]. According to the data of Oncomine database, BCL2 is markedly upregulated in the cell lines of leukemia compared to other malignancies (Fig. 1A) and is also increased in relapsed leukemia patients (Fig. 1B) and dead leukemia patients (Fig. 1C), which has allowed the use of venetoclax in the leukemia patients; meanwhile, its clinical application in AML treatment is also introduced [9, 10]. Azacitidine is a hypomethylating agent recommended as a front-line therapeutic for the elderly AML patients who are not eligible for the intensive regimen and is also approved for treating the adult patients [11]. In relapsed AML patients, these two agents are also applicable, and there are reports elucidating that the combination of venetoclax and hypomethylating agents achieves favorable responses in certain AML patient group, such as the treatment naive patients [12, 13]. As for allo-HSCT relapsed AML patients, the benefit of these two drugs is still unclear. Moreover, for eliminating the GVHD and/or relapse of AML patients receiving allo-HSCT, donor lymphocyte infusion (DLI) is a common therapy for this purpose. Hence, the potentiality of combining venetoclax, azacitidine, and DLI for treating patients with relapsed AML after allo-HSCT deserves to be investigated.

Thus, in this study, the efficacy and tolerance of venetoclax plus azacitidine and DLI in patients with relapsed AML after allo-HSCT treatment were assessed. Furthermore, we also investigated the mutated genes in our patients.

## Materials and methods

### Patients

From March 2018 to December 2019, a total of 26 patients with relapsed AML after allo-HSCT in the Hematopoietic Stem Cell Transplantation Center of Guizhou Province were recruited in this study.

The inclusion criteria were (i) diagnosed as AML and suffered from relapse after allo-HSCT; (ii) aged 16 ~ 60 years; (iii) Eastern Cooperative Oncology Group (ECOG) score of 0–2 points and life expectancy of ≥ 4 weeks; and (iv) the proportion of bone marrow (BM) blast cells at the time of relapse was less than 50% which was for the following reasons: (a) patients with high tumor burden (BM blast cells > 50%) had rapid disease progression and poor general conditions at the time of relapse. While oral venetoclax was administered at an increasing dose in patients with high tumor burden, patients might die before the evaluation time; (b) this study intended to apply this protocol in patients with low tumor burden at first to observe its efficacy, then gradually extended it to relapsed patients with high tumor burden, or even to all patients with relapsed myeloid tumors after transplantation.

The exclusion criteria were as follows: (i) patients with acute promyelocytic leukemia (APL); (ii) patients with severe arrhythmia, grade II and above cardiac dysfunction according to the New York Heart association (NYHA) standard, or cardiac ejection fraction (EF) below 45%; (iii) patients with severe pulmonary dysfunction (obstructive or restrictive ventilation disorder); (iv) patients with severe liver function impairment (over threefold higher liver function indexes (alanine transaminase, total bilirubin) than the upper limit of normal values (ULN)); (v) patients with severe renal insufficiency and over twofold higher renal function index (Cr) than ULN; (vi) patients with 24-h urinary creatinine clearance (Ccr) below 50 mL/min; (vii) patients with severe active infection; (viii) patients with clinical symptoms of brain dysfunction or severe mental illness that could not understand or follow the treatment plan; (ix) pregnant or lactating women or those prepared to get pregnant; (x) patients with other malignancies that required treatment; and (xi) previous GVHD by transplantation.

### Ethical statement

This study was approved by the Ethics Committee of The Affiliated Hospital of Guizhou Medical University and was performed in line with the Declaration of Helsinki. All participants signed informed consent forms.

### Definition of relapsed AML after allo-HSCT

Relapse of AML after allo-HSCT was defined as the occurrence of any of the following conditions: (a) the leukemia cells reappeared in peripheral blood, (b) the percentage of blast cells in bone marrow exceeded 5%, (c) positive leukemia cells were detected in a minimal residual disease, or (d) extramedullary infiltration finding.

### Treatment

In the present study, the median time to relapse after allo-HSCT (termed as duration of remission (DOR)) was 7.0 months (range: 3.2 ~ 18.4 months), and the median time to initiation of study treatment was 10 days (range: 7 ~ 20 days). All patients were treated with the venetoclax plus azacitidine and DLI. Venetoclax was given to patients by oral administration at a dose of 100 mg once a day (qd) in the first week, 200 mg qd in the second week, 300 mg qd in the third week, and a final dose at 400 mg/day as maintenance dose. Azacitidine at a dose of 75 mg/m^2^/day was administered subcutaneously from day 1 to day 5 per 28-day treatment cycle, up to 6 ~ 8 cycles. The DLI was administered on day 6, and granulocyte colony-stimulating factor (G-CSF)–mobilized peripheral blood stem cells were used in DLI. Before DLI, the G-CSF was administered for the donor subcutaneously at a dose of 10 μg/kg/day for 4 days to mobilize the HSCs, and then peripheral blood was collected on the 4th day. The median dose of mononuclear cells, CD3^+^ cells, and CD34^+^ cells for each DLI was 1.32 × 10^8^/kg (range: 0.91 to 1.54 × 10^8^/kg), 0.29 × 10^8^/kg (range: 0.12 to 0.58 × 10^8^/kg), and 1.34 × 10^6^/kg (range: 0.78 to 2.01 × 10^6^/kg), respectively. All patients did not receive cyclosporine (CSA) to prevent GVHD, and if patients had no GVHD, the DLI was repeated every 3 months.

### Follow-up and evaluation

The follow-up was conducted weekly for 6 months, while the bone marrow cytology, minimal residual disease (MRD), and chimeric rates were reviewed every month. The remission status of patients was determined with reference to the International Working Group on Acute Myeloid Leukemia [14]. The objective remission rate (ORR) in this study included complete remission with incomplete recovery (CRi) and partial remission (PR). The event-free survival (EFS) and overall survival (OS) were evaluated during the follow-up. The EFS was calculated from the initiation of the venetoclax plus azacitidine and DLI treatment to the occurrence of an event or the last follow-up. In this analysis, an event was defined as the exacerbation, progression, or death of any cause (like disease progression, GVHD, and infection) [14]. OS was calculated from the initiation of the venetoclax plus azacitidine and DLI treatment to death or the last follow-up. In addition, the incidences of GVHD, adverse events, and infections were documented.

### Genetic mutation analysis

Bone marrow samples of patients were collected before allo-HSCTS, and then bone marrow mononuclear cells were isolated and sent to Guangzhou Junruikang Biotechnology Co., Ltd. (Guangdong, China) for whole exome sequencing (WES) and bioinformatic analysis. Data analysis was conducted on two public AML gene mutation data sets (TCGA-LAML and AACR Project GENIE) [15, 16]. Afterwards, the genes shared in the two data sets were compared with those screened based on the recommendations in National Comprehensive Cancer Network Clinical (NCCN) Practice Guidelines in Oncology: Acute Myeloid Leukemia (Version 3.2020) as well as the expert consensus on the application of next-generation sequencing (NGS) in hematological neoplasms (2018) [17]. The gene mutations were classified into three levels: (1) first-class mutations, had clear diagnostic, therapeutic, and prognostic significance in hematological tumors which were reported or proved in authoritative literature, guidelines, expert consensus, or large-scale reports; (2) second-class mutations, may be associated with the disease and had database or literature support, high pathogenicity, and potential clinical significance; and (3) third-class mutations, unknown mutations of clinical significance. In addition, to better understand the impacts of these gene mutations related to AML occurrence and development on the related signaling pathways, we performed Kyoto Encyclopedia of Genes and Genomes (KEGG) enrichment analysis.

### Statistical analysis

The mean value, standard deviation (SD), median, range, count, and percentage of variables were calculated for descriptive analysis. The Kaplan–Meier (K-M) method was employed to plot the EFS and OS curves, and the differences were evaluated by the Gehan-Breslow-Wilcoxon test. Statistical analysis was performed using the SPSS 22.0 software (IBM Corp., Armonk, New York, USA). A difference of *P* < 0.05 indicated significant significance.

## Results

### Baseline characteristics

In the totally 26 AML patients, the mean age was 35.2 ± 11.4 years, and there were 15 (57.7%) males as well as 11 (42.3%) females (Table 1). The numbers of patients with ECOG score of 0, 1, and 2 were 15 (57.7%), 10 (38.5%), and 1 (3.8%), respectively. In addition, the numbers of patients with cytogenetic risk status of better, intermediate, and poor risk were 0 (0.0%), 18 (69.2%), and 8 (30.8%), respectively. Besides, the median DOR was 7.6 (range: 3.2–18.4) months. The medians of BM blasts at relapse, WBC at relapse, HGB at relapse, and platelets at relapse were 24.1 (range: 7.0–41.0) %, 12.9 (range: 0.6–38.1) × 10^9^/L, 69.0 (range: 26.0–123.0) g/L, and 68.0 (range: 56.0–101.0) × 10^9^/L, respectively. Information of the remaining characteristics are displayed in Table 1. In addition, detailed information of each patient is listed in Supplementary Table 1. Regarding the information of infection, details of anti-infection treatment could be viewed in Supplementary Table 2.

**Table 1:** Characteristics of patients with relapsed AML after allo-HSCT

Items	Patients (*N* = 26)
Age, years, mean ± SD	35.2 ± 11.4
Gender, no. (%)	
Male	15 (57.7)
Female	11 (42.3)
ECOG score, no. (%)	
0	15 (57.7)
1	10 (38.5)
2	1 (3.8)
Cytogenetics risk status, no. (%)	
Better risk	0 (0.0)
Intermediate risk	18 (69.2)
Poor risk	8 (30.8)
Number of chemotherapies before allo-HSCT, no. (%)	
2	2 (7.7)
3	13 (50.0)
4	11 (42.3)
HMA therapy before allo-HSCT, no. (%)	
2 times of azacitidine	8 (30.8)
1 time of azacitidine	1 (3.8)
2 times of decitabine	3 (11.6)
1 time of decitabine	1 (3.8)
No	13 (50.0)
Remission status before allo-HSCT, no. (%)	
PR	5 (19.2)
CR	21 (80.8)
DOR after allo-HSCT, months	
Median	7.6
Min–max	3.2–18.4
BM blasts at relapse, %	
Median	24.1
Min–max	7.0–41.0
WBC at relapse, × 10^9^/L	
Median	12.9
Min–max	0.6–38.1
HGB at relapse, g/L	
Median	69.0
Min–max	26.0–123.0
Platelets at relapse, × 10^9^/L	
Median	68.0
Min–max	56.0–101.0

*AML* acute myeloid leukemia, *Allo-HSCT* allogeneic hematopoietic stem cell transplantation, *ECOG* Eastern Cooperative Oncology Group, *HMA* hypomethylating agent, *PR* partial remission, *CR* complete remission, *DOR* duration of remission, *BM* bone marrow, *WBC* white blood cell, *HGB* hemoglobin

### Remission status and GVHD

**Fig. 2:**
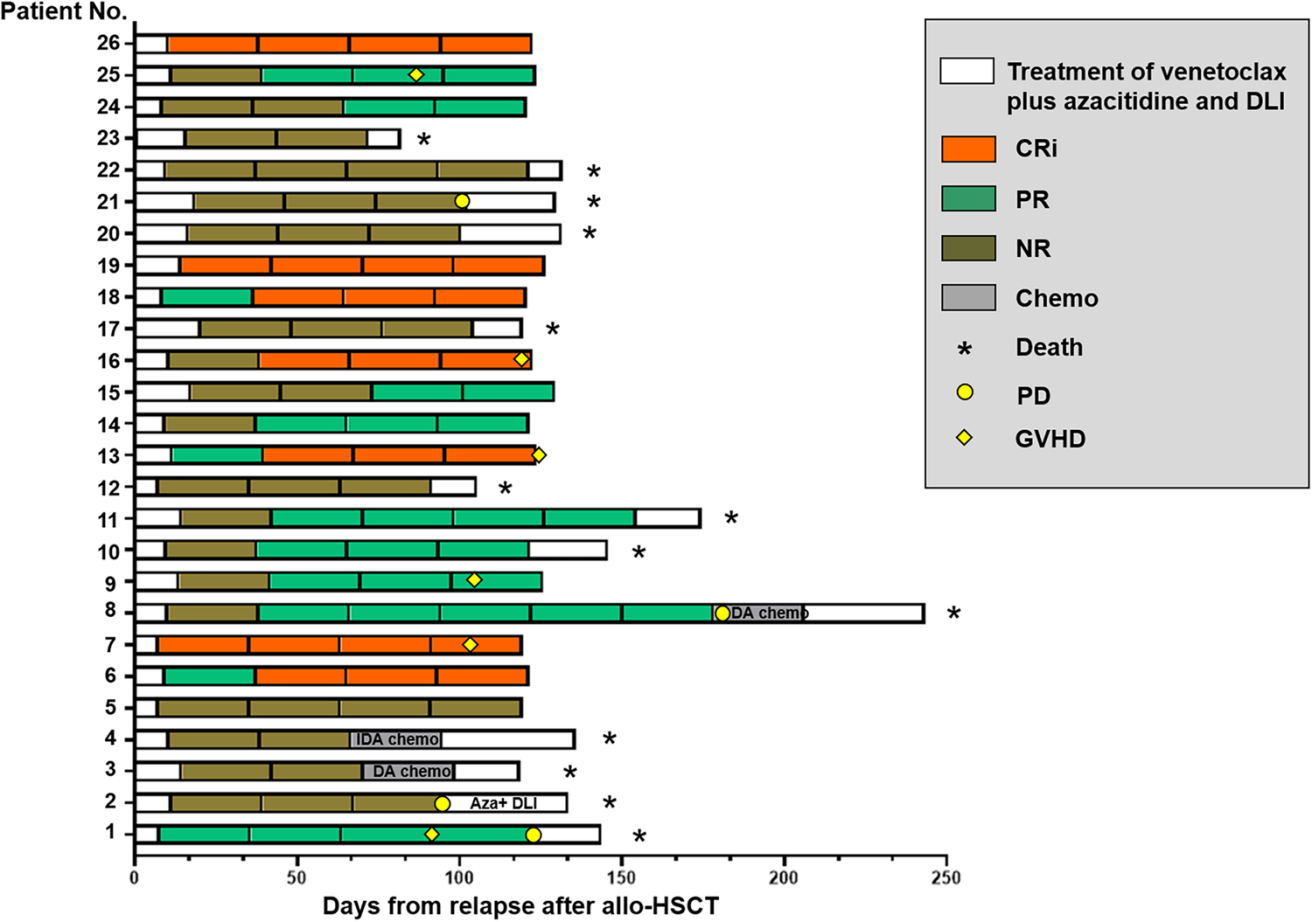
Details regarding treatment and responses of each AML patient. AML, acute myeloid leukemia; DLI, donor lymphocyte infusion; CRi, complete remission with incomplete recovery; PR, partial remission; NR, no remission; PD, progressive disease; GVHD, graft-versus-host disease

**Fig. 3:**
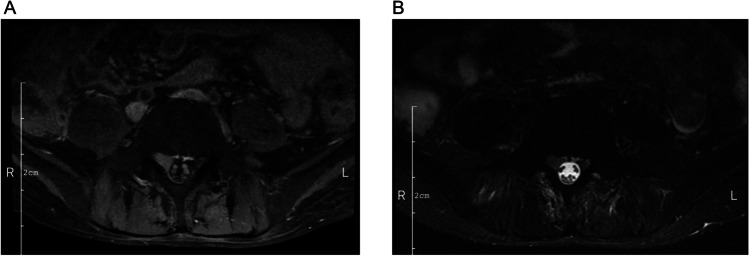
Images of one GVHD case with extramedullary infiltration. The image showing extramedullary infiltration in one patient who developed GVHD (**A**, **B**) induced by the previous allo-HSCT. GVHD, graft-versus-host disease; allo-HSCT, allogeneic hematopoietic stem cell transplantation

Precise information regarding the time of treatment courses, remission status, and GVHD of each specific patient is shown in Fig. 2. Collectively, there were 7 (26.9%), 9 (34.6%), and 10 (38.5%) patients who achieved CRi, PR, and NR, respectively (Table 2). Moreover, the median course of remission (CRi and PR) was 2 (range: 1–2). The number of patients with GVHD was 6 (23.1%). In addition, the median time to GVHD was 77 (range: 67–101) days. Moreover, a patient relapsed despite of GVHD after transplantation, with the initial manifestation of extramedullary infiltration (Fig. 3A–B), and no GVHD was induced after DLI.

**Table 2:** Summary of treatment courses, remission status, and GVHD

Items	Patients (*N* = 26)
Remission status, no. (%)	
CRi	7 (26.9)
PR	9 (34.6)
NR	10 (38.5)
Treatment courses, no. (%)	
At least 2 courses	26 (100.0)
More than 4 courses	16 (61.5)
Course of remission (CRi or PR)	
Median	2
Min–max	1–3
GVHD, no. (%)	
Total	6 (23.1)
Grade II	6 (23.1)
Time to GVHD (days)	
Median	77
Min–max	67–101

*GVHD* graft-versus-host disease, *CRi* complete remission with incomplete recovery, *PR* partial remission, *NR* no remission

### Survival profile

**Fig. 4:**
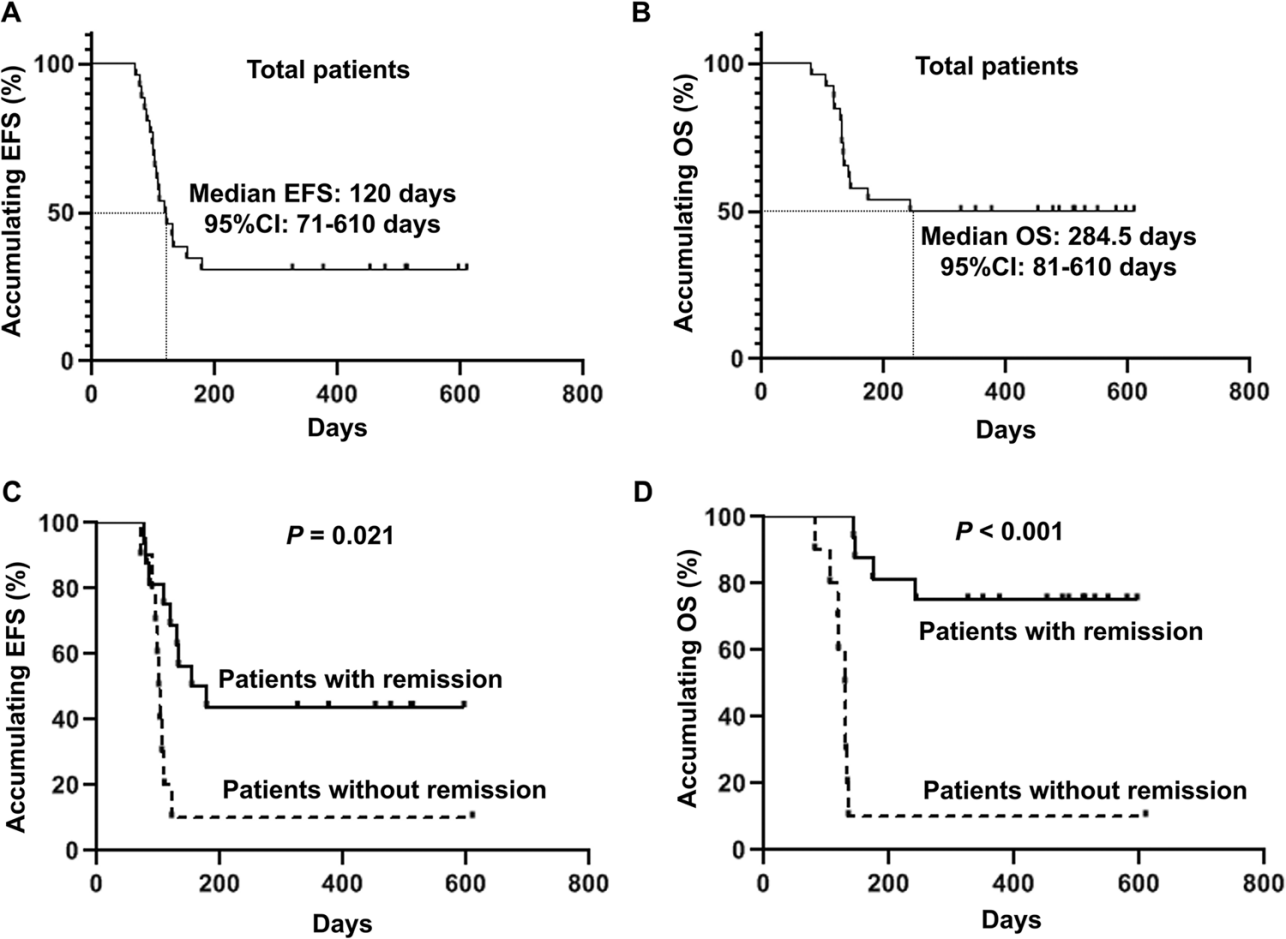
Survival analysis. The EFS (**A**) and OS (**B**) in AML patients post treatment and the correlation of EFS (**C**) as well as OS (**D**) with remission. EFS, event-free survival; OS, overall survival; AML, acute myeloid leukemia

Post treatment of venetoclax plus azacitidine and DLI, the median EFS (Fig. 4A) and OS (Fig. 4B) in the total patients were 120 (95% CI: 71–610) days and 284.5 (95% CI: 81–610) days, respectively. Moreover, the EFS was more favorable in patients with remission post treatment compared to patient without remission (*P* = 0.021) (Fig. 4C), and the OS was also longer in patients with remission compared to patients without remission (*P* < 0.001) (Fig. 4D).

### Adverse events

During and post treatment of venetoclax plus azacitidine and DLI, adverse events of the hematologic system occurred in all the patients, among which agranulocytosis and thrombocytopenia cases were all at grade III or grade IV, and 53.8% of the anemia cases were at grade III/IV (Table 3). Furthermore, as to digestive system, the prevalence of nausea and vomiting, dental ulcer, hyperbilirubinemia, elevated liver enzymes, and diarrhea were 42.3%, 23.1%, 15.4%, 11.5%, and 7.7%, respectively; additionally, most of these digestive system adverse events were mild. As for the urogenital system adverse events, the percentages of hyperkalemia and hematuresis were 11.5% and 3.8%, and no grade III/IV adverse events were found. In terms of the respiratory system, the percentages of fever, rash, and dyspnea were 100.0%, 46.2%, and 15.4%, respectively; and the portions of grade III/IV fever and dyspnea were 57.7 and 11.5%, respectively. As to the cardiovascular system, related adverse events were rare, and no grade III/IV cardiovascular system adverse events were discovered.

**Table 3:** Adverse events

Adverse events	Total adverse events	Grade III/IV adverse events
Hematologic system		
Agranulocytosis	26 (100.0)	26 (100.0)
Anemia	26 (100.0)	149 (53.8)
Thrombocytopenia	26 (100.0)	26 (100.0)
Digestive system		
Nausea and vomiting	11 (42.3)	2 (7.7)
Dental ulcer	6 (23.1)	1 (3.8)
Hyperbilirubinemia	4 (15.4)	0 (0.0)
Elevated liver enzymes	3 (11.5)	0 (0.0)
Diarrhea	2 (7.7)	0 (0.0)
Urogenital system		
Hyperkalemia	3 (11.5)	0 (0.0)
Hematuresis	1 (3.8)	0 (0.0)
Respiratory system		
Fever	26 (100.0)	15 (57.7)
Rash	12 (46.2)	0 (0.0)
Dyspnea	4 (15.4)	3 (11.5)
Cardiovascular system		
Peripheral edema	3 (11.5)	0 (0.0)
Headache	2 (7.7)	0 (0.0)

### Mutated genes and their correlations with survival profile

**Fig. 5:**
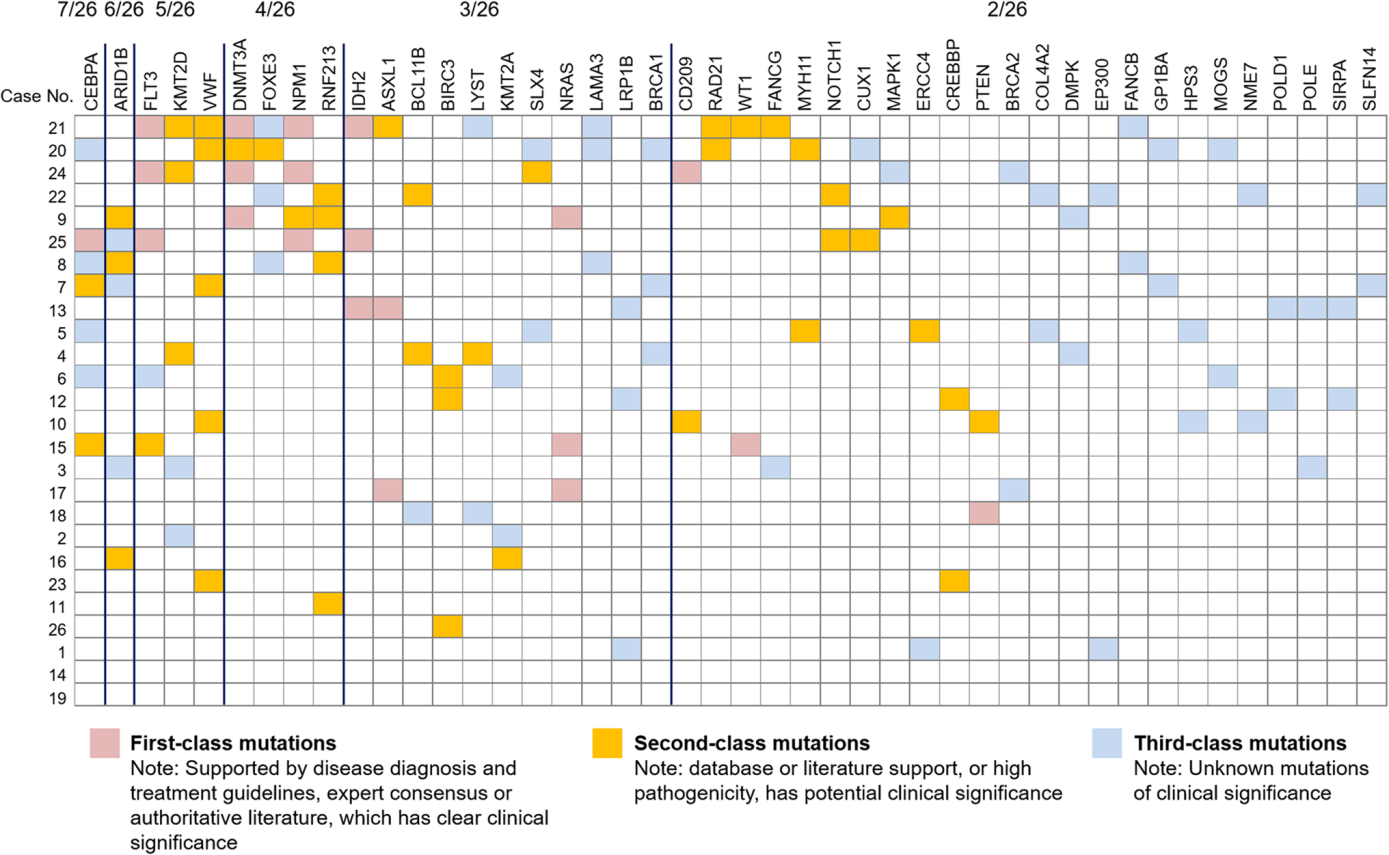
Overall mutated genes in patients with relapsed AML after allo-HSCT. AML, acute myeloid leukemia; allo-HSCT, allogeneic hematopoietic stem cell transplantation

**Fig. 6:**
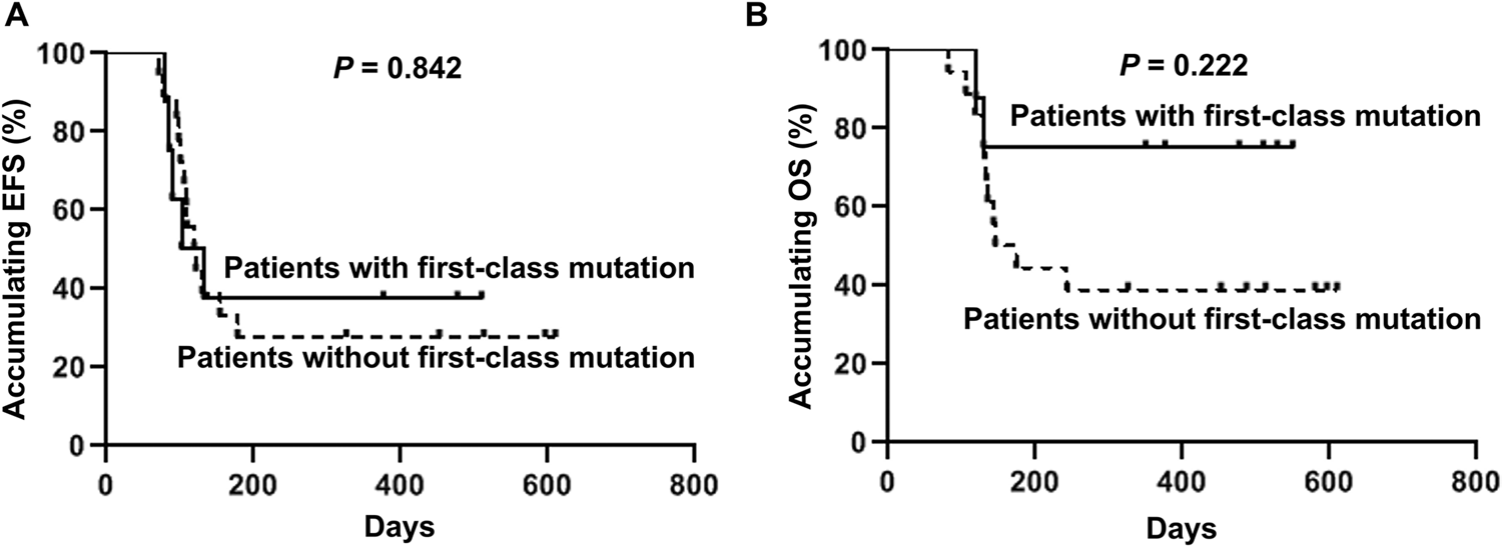
Correlation of first-class mutation with survival. The correlation of first-class mutation with EFS (**A**) and OS (**B**) in patients with relapsed AML after allo-HSCT. EFS, event-free survival; OS, overall survival; AML, acute myeloid leukemia; allo-HSCT, allogeneic hematopoietic stem cell transplantation

Overall information of mutated genes in the total patients are shown in Fig. 5, which included first-class, second-class, and third-class mutated genes. Then according to the mutated genes found in our study, patients were divided into two groups based on whether they had first-class mutation, and the analyses disclosed that no difference regarding EFS (*P* = 0.842) (Fig. 6A) or OS (*P* = 0.222) (Fig. 6B) was found between patients with first-class mutation and patients without first-class mutation.

### Enrichment analysis of mutated genes

**Fig. 7:**
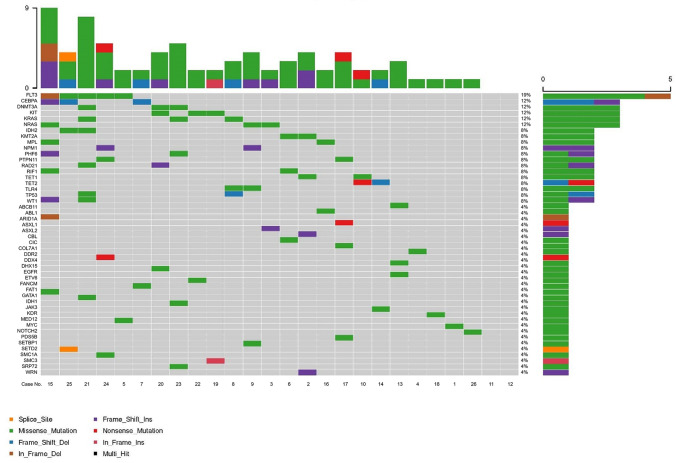
Frequencies of mutated genes. AML, acute myeloid leukemia

**Fig. 8:**
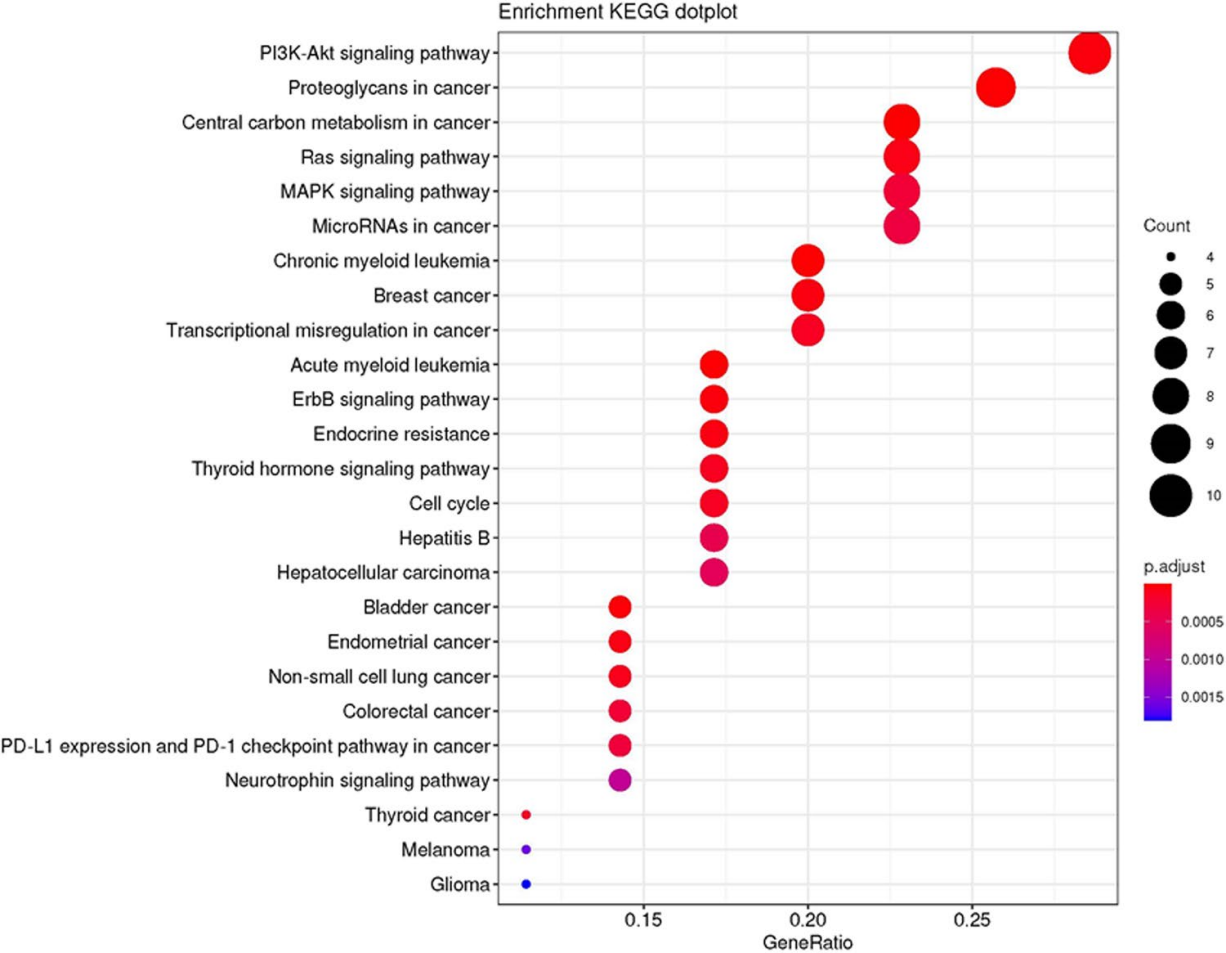
Enrichment analysis of the mutated genes. AML, acute myeloid leukemia; KEGG, Kyoto Encyclopedia of Genes and Genomes

**Fig. 9:**
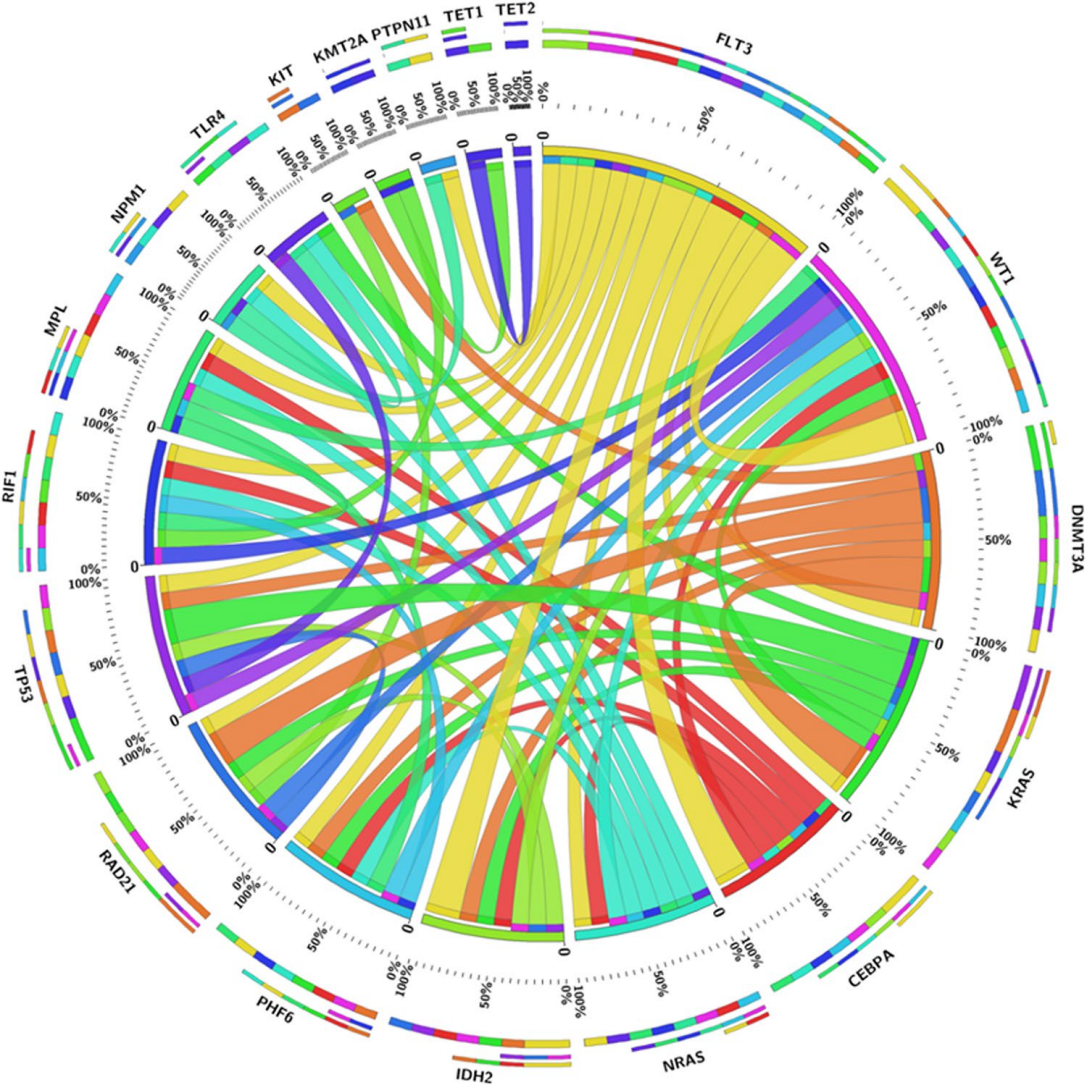
Regulatory network of the mutated genes. AML, acute myeloid leukemia

Among all the mutated genes, there were several mutations exhibited relatively high prevalence, including the FLT3 mutation with a prevalence of 19% and CEBPA, DNMT3A, KIT, KRAS, and NRAS mutations with a prevalence of 12% (Fig. 7). The further enrichment analysis of all the mutated genes revealed that they were markedly enriched in multiple AML-related signaling pathways, which consisted of PI3K-Akt signaling pathway, Ras signaling pathway, MAPK signaling pathways, etc., and the known chronic myeloid leukemia-related signaling pathways as well as AML-related signaling pathways (Fig. 8). In addition, the interactions among all the mutated genes were displayed in a circos plot (Fig. 9).

## Discussion

Relapsed disease remains to be a crucial unsolved issue in AML, even after the treatment of transplantation, and it is still a common phenomenon. Relapsed AML, no matter the previous treatments, is difficult to manage which often requires a team of clinicians for decision-making; therefore, exploring novel and more effective therapeutic regimens has never been stopped. In the present study, we tried to investigate the efficacy and safety of venetoclax plus azacitidine and DLI for patients with relapsed AML after allo-HSCT, and our results revealed that (1) post treatment of venetoclax plus azacitidine and DLI, the rates of CRi and PR were 26.9% and 34.6%, respectively; in terms of survival profile, median EFS was 120 days and median OS was 284.5 days, and EFS and OS were both more favorable in patients with remission; (2) the most common adverse events post treatment were agranulocytosis, anemia, and thrombocytopenia, and no serious adverse events were found in our study; (3) subsequently, 49 mutated genes were detected by the next-generation sequencing and were categorized to first-, second-, and third-class mutations; in addition, the first-class mutations were not correlated with EFS or OS.

Efficacy of venetoclax plus azacitidine and DLI for patients with relapsed AML after allo-HSCT is largely unknown; however, the combination of two or single use of these therapies is plentifully reported. Such as, in a patient group of relapsed and refractory AML patients and other myeloid malignancies (more than 90% cases being AML), the treatment of venetoclax with other therapies (hypomethylating agents or low-dose cytarabine) achieves an objective response of 21% and a median survival time of 3.0 months (range: 0.5–8.0 months) [18]. Another study retrospectively analyzes the data from 11 centers and finds that in AML patients who are relapsed/refractory post intensive chemotherapy, treatment with venetoclax combined with hypomethylating agents leads to 76% neutrophil recovery and 59% platelet count recovery in patients who survive for over two cycles of treatments [19]. A single-arm study using azacitidine plus nivolumab for relapsed/refractory AML patients elucidates that post treatment, the overall response rate is 33% and the CR rate is 22%; in addition, SD rate is 9% [20]. Moreover, another study illuminates that in AML patients who relapse post allograft, treatment of 5-azacytidine combined with DLI achieves a CR rate of 9%, more importantly, among whom 2 patients maintain to be CR for more than 2 years; and 44% of the patients present with temporary disease control; besides, the median survival is 108 days [21]. In our study, we found that in patients with relapsed AML after allo-HSCT, the treatment of venetoclax plus azacitidine and DLI achieved CRi and PR of 26.9% and 34.6%; in terms of survival profile, the median EFS was 120 days and median OS was 284.5 days, and EFS and OS were both more favorable in patients with remission. These all implied that combined therapy of venetoclax plus azacitidine and DLI was efficient in patients with relapsed AML after allo-HSCT.

Acceptable tolerance is required for AML patients’ treatment, especially for those who relapse from multiple therapies, including allo-HSCT. The three treatments in our study, venetoclax, azacitidine, and DLI, are reported to be tolerable in relapsed AML patients, both in combination with other therapies or using alone. A previous study reports that in AML patients who relapse after allo-HSCT and then treated with venetoclax plus DLI, most of the patients can tolerate the post-treatment adverse events without admissions to the hospital [22]. In addition, a phase I/II study reveals that using azacitidine and gemtuzumab ozogamicin (GO) for relapsed AML patients, post treatment, there does not exist any dose-limiting toxicities (75 mg/m^2^ daily for 6 consecutive days, followed by GO 6 mg/m^2^ on days 7 and 21) or hepatic sinusoidal obstructive syndrome [23]. Another phase I study illuminates that DLI followed by azacitidine for treating AML patients who relapse after allo-HSCT, no cases of grade III/IV GVHD are found during the follow-up (with median follow-up time of 5.2 months), and there are also no patients who die due to GVHD [24]. As for the tolerance of the combined treatment in our study, the most common adverse events were granulocytosis, anemia, and thrombocytopenia, with most of them being grade III/IV. Most importantly, no serious adverse events were discovered in our patients.

Genetic abnormality has important impact on AML prognosis, not only the well-known NCCN risk stratification involved cytogenetic abnormity and molecule genes, but also other recent identified prognostic genes [25–27]. Therefore, we then detected the gene mutations in our patients and found 49 mutated genes which were classified as first-, second-, and third-class mutations. Then further analysis revealed that there was no difference regarding EFS and OS between patients with first-class mutation and patients without first-class mutation. As known, the first-class mutations included prognostic genes such as CEBPA, FLT3, DNMT3A, NPM1, and RNF213; they are previously observed to correlate with prognosis of AML [28–30]. However, our study indicated that the mutated genes might not be correlated with survival in patients with relapsed AML after allo-HSCT, which might result from the reduced small sample size in our study; this presumption needed more validation by future studies.

As for the rational of venetoclax plus azacitidine and DLI regimen in our study, post-HSCT AML patients are often complicated with anemia and thrombocytopenia, so a proportion of relapsed patients (low bone marrow blast count acute myeloid leukemia) are commonly treated by less aggressive therapy. According to NCCN guideline and several articles for less aggressive therapy of AML, venetoclax plus hypomethylating agents (such as azacitidine) is recommended [18, 31]. Besides, azacytidine and decitabine (as hypomethylating agents) are commonly used drug not only before but also after transplantation, so for some high-risk patients, they are used both before and after transplantation. In addition, DLI is the basic treatment for relapsed AML post transplantation [32]. Therefore, venetoclax plus azacitidine and DLI regimen is used in our study.

Furthermore, the enrolled patients were from year 2018–2019; at that time, the clinical experience of venetoclax administration was very limited in China; therefore, we increased venetoclax dose every week (at a dose of 100 mg once a day (qd) in the first week, 200 mg qd in the second week, 300 mg qd in the third week, and a final dose at 400 mg/day as maintenance dose) to explore the experience instead of every 2 days or every 3–5 days [22, 33]. Meanwhile, the delayed recovery of hemogram was another reason we increased the venetoclax dose slowly. Furthermore, due to that the analyzed patients realized CR by bone marrow examination and showed no MRD, but the majority of them lacked complete recovery of WBC and platelets (which might be due to maintenance use of venetoclax), so CRi was used for data accuracy.

There were several limitations in the present study that should not be ignored, which included that the sample size was small, which may interfere with the statistical power. In addition, the follow-up duration was also relatively short. Last, the AML patients in our study were all with a relatively younger age (less than 60 years), which might block the potential utilization of our results in the elderly patients.

In summary, venetoclax plus azacitidine and DLI is efficient and tolerant in treating patients with relapsed AML after allo-HSCT, implying this combined therapy as a potential treatment option in the studied patients.

## Supplementary Information

Below is the link to the electronic supplementary material.

Supplementary file1 (DOCX 22 KB)

## References

[1] Saleh K, Khalifeh-Saleh N, Kourie HR (2020). Acute myeloid leukemia transformed to a targetable disease. Future Oncol.

[2] Pelcovits A (2013). Niroula R (2020) Acute myeloid leukemia: a review. R I Med J.

[3] Ferrara F, Lessi F, Vitagliano O, Birkenghi E, Rossi G (2019) Current therapeutic results and treatment options for older patients with relapsed acute myeloid leukemia. Cancers (Basel) 11 (2). 10.3390/cancers11020224 10.3390/cancers11020224 PMC6406399 30769877

[4] Bryan JC, Jabbour EJ (2015). Management of relapsed/refractory acute myeloid leukemia in the elderly: current strategies and developments. Drugs Aging.

[5] Hutter-Kronke ML, Fiedler W, Kundgen A, Krauter J, von Lilienfeld-Toal M, Dohner H, Schlenk RF (2019). Continuous high dosing of lenalidomide in relapsed, refractory or older newly diagnosed acute myeloid leukemia patients not suitable for other treatment options - results from a phase I study. Haematologica.

[6] Arfons LM, Tomblyn M, Rocha V, Lazarus HM (2009). Second hematopoietic stem cell transplantation in myeloid malignancies. Curr Opin Hematol.

[7] Nemkov T, D'Alessandro A, Hansen KC (2015). Three-minute method for amino acid analysis by UHPLC and high-resolution quadrupole orbitrap mass spectrometry. Amino Acids.

[8] Chan SM, Thomas D, Corces-Zimmerman MR, Xavy S, Rastogi S, Hong WJ, Zhao F, Medeiros BC, Tyvoll DA, Majeti R (2015). Isocitrate dehydrogenase 1 and 2 mutations induce BCL-2 dependence in acute myeloid leukemia. Nat Med.

[9] Wei Y, Xiong X, Li X, Lu W, He X, Jin X, Sun R, Lyu H, Yuan T, Sun T, Zhao M (2021). Low-dose decitabine plus venetoclax is safe and effective as post-transplant maintenance therapy for high-risk acute myeloid leukemia and myelodysplastic syndrome. Cancer Sci.

[10] Andreani G, Dragani M, Serra A, Nicoli P, De Gobbi M, Cilloni D (2019). Venetoclax plus decitabine induced complete remission with molecular response in acute myeloid leukemia relapsed after hematopoietic stem cell transplantation. Am J Hematol.

[11] Scott LJ (2016). Azacitidine: a review in myelodysplastic syndromes and acute myeloid leukaemia. Drugs.

[12] DiNardo CD, Pratz K, Pullarkat V, Jonas BA, Arellano M, Becker PS, Frankfurt O, Konopleva M, Wei AH, Kantarjian HM, Xu T, Hong WJ, Chyla B, Potluri J, Pollyea DA, Letai A (2019). Venetoclax combined with decitabine or azacitidine in treatment-naive, elderly patients with acute myeloid leukemia. Blood.

[13] DiNardo CD, Jonas BA, Pullarkat V, Thirman MJ, Garcia JS, Wei AH, Konopleva M, Dohner H, Letai A, Fenaux P, Koller E, Havelange V, Leber B, Esteve J, Wang J, Pejsa V, Hajek R, Porkka K, Illes A, Lavie D, Lemoli RM, Yamamoto K, Yoon SS, Jang JH, Yeh SP, Turgut M, Hong WJ, Zhou Y, Potluri J, Pratz KW (2020). Azacitidine and venetoclax in previously untreated acute myeloid leukemia. N Engl J Med.

[14] Cheson BD, Bennett JM, Kopecky KJ, Buchner T, Willman CL, Estey EH, Schiffer CA, Doehner H, Tallman MS, Lister TA, Lo-Coco F, Willemze R, Biondi A, Hiddemann W, Larson RA, Lowenberg B, Sanz MA, Head DR, Ohno R, Bloomfield CD, International Working Group for Diagnosis SoRCTO, Reporting Standards for Therapeutic Trials in Acute Myeloid L (2003). Revised recommendations of the International Working Group for diagnosis, standardization of response criteria, treatment outcomes, and reporting standards for therapeutic trials in acute myeloid leukemia. J Clin Oncol.

[15] Ley TJ, Miller C, Ding L, Raphael BJ, Mungall AJ, Robertson A, Hoadley K, Triche TJ, Laird PW, Baty JD, Fulton LL, Fulton R, Heath SE, Kalicki-Veizer J, Kandoth C, Klco JM, Koboldt DC, Kanchi KL, Kulkarni S, Lamprecht TL, Larson DE, Lin L, Lu C, McLellan MD, McMichael JF, Payton J, Schmidt H, Spencer DH, Tomasson MH, Wallis JW, Wartman LD, Watson MA, Welch J, Wendl MC, Ally A, Balasundaram M, Birol I, Butterfield Y, Chiu R, Chu A, Chuah E, Chun HJ, Corbett R, Dhalla N, Guin R, He A, Hirst C, Hirst M, Holt RA, Jones S, Karsan A, Lee D, Li HI, Marra MA, Mayo M, Moore RA, Mungall K, Parker J, Pleasance E, Plettner P, Schein J, Stoll D, Swanson L, Tam A, Thiessen N, Varhol R, Wye N, Zhao Y, Gabriel S, Getz G, Sougnez C, Zou L, Leiserson MD, Vandin F, Wu HT, Applebaum F, Baylin SB, Akbani R, Broom BM, Chen K, Motter TC, Nguyen K, Weinstein JN, Zhang N, Ferguson ML, Adams C, Black A, Bowen J, Gastier-Foster J, Grossman T, Lichtenberg T, Wise L, Davidsen T, Demchok JA, Shaw KR, Sheth M, Sofia HJ, Yang L, Downing JR, Eley G, Cancer Genome Atlas Research N (2013). Genomic and epigenomic landscapes of adult de novo acute myeloid leukemia. N Engl J Med.

[16] Papaemmanuil E, Gerstung M, Bullinger L, Gaidzik VI, Paschka P, Roberts ND, Potter NE, Heuser M, Thol F, Bolli N, Gundem G, Van Loo P, Martincorena I, Ganly P, Mudie L, McLaren S, O'Meara S, Raine K, Jones DR, Teague JW, Butler AP, Greaves MF, Ganser A, Dohner K, Schlenk RF, Dohner H, Campbell PJ (2016). Genomic classification and prognosis in acute myeloid leukemia. N Engl J Med.

[17] 汝昆 (2019) 《二代测序技术在血液肿瘤中的应用中国专家共识(2018年版)》解读. 临床血液学杂志 32 (03):20–22+26

[18] DiNardo CD, Rausch CR, Benton C, Kadia T, Jain N, Pemmaraju N, Daver N, Covert W, Marx KR, Mace M, Jabbour E, Cortes J, Garcia-Manero G, Ravandi F, Bhalla KN, Kantarjian H, Konopleva M (2018). Clinical experience with the BCL2-inhibitor venetoclax in combination therapy for relapsed and refractory acute myeloid leukemia and related myeloid malignancies. Am J Hematol.

[19] Ganzel C, Ram R, Gural A, Wolach O, Gino-Moor S, Vainstein V, Nachmias B, Apel A, Koren-Michowitz M, Pasvolsky O, Yerushalmi R, Danylesko I, Cohen Y, Peretz G, Moshe Y, Zektser M, Yeganeh S, Rowe JM, Ofran Y (2020). Venetoclax is safe and efficacious in relapsed/refractory AML. Leuk Lymphoma.

[20] Daver N, Garcia-Manero G, Basu S, Boddu PC, Alfayez M, Cortes JE, Konopleva M, Ravandi-Kashani F, Jabbour E, Kadia T, Nogueras-Gonzalez GM, Ning J, Pemmaraju N, DiNardo CD, Andreeff M, Pierce SA, Gordon T, Kornblau SM, Flores W, Alhamal Z, Bueso-Ramos C, Jorgensen JL, Patel KP, Blando J, Allison JP, Sharma P, Kantarjian H (2019). Efficacy, safety, and biomarkers of response to azacitidine and nivolumab in relapsed/refractory acute myeloid leukemia: a nonrandomized, open-label, phase II study. Cancer Discov.

[21] Steinmann J, Bertz H, Wasch R, Marks R, Zeiser R, Bogatyreva L, Finke J, Lubbert M (2015). 5-Azacytidine and DLI can induce long-term remissions in AML patients relapsed after allograft. Bone Marrow Transplant.

[22] Amit O, On YB, Perez G, Shargian-Alon L, Yeshurun M, Ram R (2021). Venetoclax and donor lymphocyte infusion for early relapsed acute myeloid leukemia after allogeneic hematopoietic cell transplantation. A retrospective multicenter trial Ann Hematol.

[23] Medeiros BC, Tanaka TN, Balaian L, Bashey A, Guzdar A, Li H, Messer K, Ball ED (2018) A phase I/II trial of the combination of azacitidine and gemtuzumab ozogamicin for treatment of relapsed acute myeloid leukemia. Clin Lymphoma Myeloma Leuk 18 (5):346–352 e345. 10.1016/j.clml.2018.02.017 10.1016/j.clml.2018.02.017 29572158

[24] Ghobadi A, Choi J, Fiala MA, Fletcher T, Liu J, Eissenberg LG, Abboud C, Cashen A, Vij R, Schroeder MA, Pusic I, Stockerl-Goldstein K, Jacoby M, Uy G, DiPersio J, Westervelt P (2016). Phase I study of azacitidine following donor lymphocyte infusion for relapsed acute myeloid leukemia post allogeneic stem cell transplantation. Leuk Res.

[25] Pollyea DA, Bixby D, Perl A, Bhatt VR, Altman JK, Appelbaum FR, de Lima M, Fathi AT, Foran JM, Gojo I, Hall AC, Jacoby M, Lancet J, Mannis G, Marcucci G, Martin MG, Mims A, Neff J, Nejati R, Olin R, Percival ME, Prebet T, Przespolewski A, Rao D, Ravandi-Kashani F, Shami PJ, Stone RM, Strickland SA, Sweet K, Vachhani P, Wieduwilt M, Gregory KM, Ogba N, Tallman MS (2021) NCCN guidelines insights: acute myeloid leukemia, version 2.2021. J Natl Compr Canc Netw 19 (1):16–27. 10.6004/jnccn.2021.0002 10.6004/jnccn.2021.0002 33406488

[26] Wu M, Guo ZW, Huang GN, Ye YB (2021). Features and impacts on the prognosis of gene mutations in patients with acute myeloid leukemia. Neoplasma.

[27] Lai Y, Sheng L, Wang J, Zhou M, OuYang G (2021). A novel 85-gene expression signature predicts unfavorable prognosis in acute myeloid leukemia. Technol Cancer Res Treat.

[28] Xu X, Cai W, Cai P, Zhang L, Yao H, Zhang T, Shen H, Chen S (2021). Prognostic nomogram for acute myeloid leukemia patients with biallelic CEBPA mutations. Front Oncol.

[29] Onate G, Bataller A, Garrido A, Hoyos M, Arnan M, Vives S, Coll R, Tormo M, Sampol MA, Escoda L, Salamero O, Garcia A, Bargay J, Aljarilla A, Nomdedeu JF, Esteve J, Sierra J, Pratcorona M (2021). Prognostic impact of DNMT3A mutation in acute myeloid leukemia with mutated NPM1. Blood Adv.

[30] Rehman A, Akram AM, Chaudhary A, Sheikh N, Hussain Z, Alsanie WF, Rehman RA, Hameed N, Saleem T, Zafar A, Absar M, Iqbal Z, Alhazmi A, Baeshen HA, Mohammedsaleh ZM, Qamer S, Sayed S, Gaber A (2021). RUNX1 mutation and elevated FLT3 gene expression cooperates to induce inferior prognosis in cytogenetically normal acute myeloid leukemia patients. Saudi J Biol Sci.

[31] Aldoss I, Yang D, Aribi A, Ali H, Sandhu K, Al Malki MM, Mei M, Salhotra A, Khaled S, Nakamura R, Snyder D, O'Donnell M, Stein AS, Forman SJ, Marcucci G, Pullarkat V (2018). Efficacy of the combination of venetoclax and hypomethylating agents in relapsed/refractory acute myeloid leukemia. Haematologica.

[32] Loren AW, Porter DL (2008). Donor leukocyte infusions for the treatment of relapsed acute leukemia after allogeneic stem cell transplantation. Bone Marrow Transplant.

[33] Samra B, Konopleva M, Isidori A, Daver N, DiNardo C (2020). Venetoclax-based combinations in acute myeloid leukemia: current evidence and future directions. Front Oncol.

