# Supplementary material for: Venetoclax plus azacitidine and donor lymphocyte infusion in treating acute myeloid leukemia patients who relapse after allogeneic hematopoietic stem cell transplantation
Source: Ann Hematol. 2021 Sep 27;101(1):119–30. doi: 10.1007/s00277-021-04674-x (PMC8720738; doi:10.1007/s00277-021-04674-x)
Supplement: Supplementary file 1 — Supplementary file1 (DOCX 22 KB) [file 277_2021_4674_MOESM1_ESM.docx]

**Supplementary Table 1.** Detailed information of patients

| No. | Age (years) | Gender | ECOG score | Cytogenetics risk | Before allo-HSCT | | | | HLA | DOR (months) after allo-HSCT | Blast count (%) | Remission status | GVHD |
| --- | --- | --- | --- | --- | --- | --- | --- | --- | --- | --- | --- | --- | --- |
|  |  |  |  |  | Chemo times | HMA | Remission status | Preconditioning |  |  |  |  |  |
| 1 | 48 | Male | 0 | Intermediate | 4 | 2Aza | CR | BuCY+ATG+Aza | HLA 6/12; O/A | 4.9 | 23.0 | PR | I-GVHD |
| 2 | 45 | Male | 1 | Poor | 3 | 2Dec | CR | BuCY+ATG+Dec | HLA 10/12; A/A | 4.2 | 22.5 | NR | No |
| 3 | 34 | Female | 1 | Intermediate | 4 | No | CR | BuCY+ATG+Aza | HLA 7/12; AB/B | 12.4 | 24.2 | NR | No |
| 4 | 39 | Male | 1 | Poor | 2 | Dec | CR | BuCY+Dec | HLA 12/12; B/B | 3.2 | 11.2 | NR | No |
| 5 | 27 | Female | 1 | Intermediate | 4 | No | CR | BuCY+ATG+Aza | HLA 6/12; A/A | 3.2 | 26.0 | NR | No |
| 6 | 31 | Male | 2 | Intermediate | 3 | No | CR | BuCY+ATG+Aza | HLA 9/12; O/A | 18.4 | 22.0 | CRi | No |
| 7 | 41 | Female | 0 | Poor | 3 | 2Aza | PR | Bu4CY+ATG+Aza | HLA 11/12; O/O | 13.6 | 21.0 | CRi | II-GVHD |
| 8 | 20 | Female | 0 | Poor | 2 | No | PR | Bu4CY+ATG+Aza | HLA 6/12; AB/AB | 6.5 | 26.0 | PR | No |
| 9 | 39 | Female | 0 | Intermediate | 4 | No | CR | BuCY | HLA 12/12; B/O | 16.7 | 23.4 | PR | II-GVHD |
| 10 | 16 | Male | 1 | Intermediate | 4 | No | CR | BuCY+ATG+Aza | HLA 6/12; A/A | 17.1 | 22.0 | PR | No |
| 11 | 44 | Male | 0 | Intermediate | 3 | 2Aza | CR | BuCY+ATG+Aza | HLA 8/12; O/O | 17.1 | 38.0 | PR | No |
| 12 | 21 | Male | 0 | Intermediate | 3 | No | PR | Bu4CY+ATG+Aza | HLA 10/12; O/B | 3.8 | 39.0 | NR | No |
| 13 | 41 | Female | 1 | Intermediate | 4 | 2Aza | CR | BuCY+ATG+Aza | HLA 7/12; A/A | 13.0 | 26.4 | CRi | II-GVHD |
| 14 | 54 | Male | 0 | Poor risk | 4 | 2Aza | CR | BuCY+ATG+Aza | HLA 6/12; O/AB | 13.0 | 23.0 | PR | No |
| 15 | 28 | Male | 0 | Poor risk | 3 | No | CR | BuCY+Aza | HLA 12/12; AB/AB | 8.2 | 11.0 | PR | No |
| 16 | 23 | Female | 1 | Intermediate | 3 | No | CR | BuCY | HLA 12/12; B/O | 10.0 | 41.0 | CRi | III-GVHD |
| 17 | 39 | Male | 0 | Intermediate | 3 | 2Aza | CR | BuCY+ATG+Aza | HLA 6/12; A/A | 3.4 | 28.0 | NR | II-GVHD |
| 18 | 23 | Female | 1 | Intermediate | 4 | No | CR | BuCY+ATG+Aza | HLA 7/12; B/A | 4.8 | 14.0 | CRi | No |
| 19 | 51 | Male | 0 | Intermediate | 4 | 2Aza | CR | BuCY+ATG+Aza | HLA 8/12; A/O | 7.0 | 24.0 | CRi | No |
| 20 | 16 | Male | 1 | Intermediate | 4 | No | CR | BuCY+ATG+Aza | HLA 8/12; A/AB | 6.3 | 28.0 | NR | No |
| 21 | 46 | Male | 0 | Intermediate | 3 | 2Aza | CR | BuCY+Aza | HLA 12/12; A/A | 10.8 | 36.0 | NR | No |
| 22 | 23 | Female | 0 | Intermediate | 4 | No | CR | BuCY+ATG | HLA 6/12; A/A | 10.0 | 19.0 | NR | No |
| 23 | 44 | Male | 0 | Intermediate | 3 | 2Dec | CR | BuCY+Dec | HLA 12/12; B/B | 5.1 | 22.0 | NR | No |
| 24 | 47 | Male | 0 | Poor | 3 | 2Dec | PR | Bu4CY+ATG+Aza | HLA 10/12; O/O | 5.6 | 7.0 | PR | No |
| 25 | 29 | Female | 0 | Intermediate | 3 | No | CR | BuCY+ATG | HLA 6/12; B/B | 7.0 | 23.1 | PR | II-GVHD |
| 26 | 45 | Female | 1 | Poor | 3 | Aza | PR | Bu4CY+Aza | HLA 12/12; O/B | 3.4 | 8.0 | CRi | No |

ECOG: Eastern Cooperative Oncology Group; Allo-HSCT: allogeneic hematopoietic stem cell transplantation; HMA, hypomethylating agent; Aza, Azacitidine (“2Aza” means two times of Aza treatment); Dec, decitabine (“2Dec” means two times of Dec treatment); BuCY, busulfan and cyclophosphamide; ATG, antithymocyte globulin; HLA, human leukocyte antigen; DOR: duration of remission; GVHD, graft-versus-host disease; CR: complete remission; PR: partial remission; CRi, complete remission with incomplete recovery; NR, no remission.
